# The challenges of diagnosing idiopathic ovarian vein thrombosis: Case report

**DOI:** 10.1016/j.ijscr.2019.04.039

**Published:** 2019-05-03

**Authors:** Muntadhar Mahdi Alalqam, Riyadh Al Abbas, Abdaljaleel Shaker Abualsaud, Abdullah Saleh AlQattan, Fatimah Almabyouq

**Affiliations:** a Imam Abdulrahman bin Faisal University, King Fahad University Hospital, Saudi Arabia; b Dammam Medical Complex Hospital, Saudi Arabia; c Imam Abdulrahman bin Faisal University, Department of General Surgery, King Fahad University Hospital, Saudi Arabia

**Keywords:** Abdominal pain, Acute abdomen, Idiopathic, Ovarian vein thrombosis

## Abstract

**Introduction:**

Abdominal pain is a common clinical challenge presenting to the emergency department. The challenge lies in the wide differentials particularly in females due to the addition of potential gynecological conditions. Ovarian vein thrombosis (OVT) is one of the uncommon differentials requiring rapid recognition & treatment to avoid serious complications or even death.

**Case presentation:**

We report a 42-year-old healthy female, post normal vaginal delivery 1- year prior to presentation. She presented with left iliac fossa and periumbilical pain for 1-day with no other symptoms. She was vitally stable. Abdominal & pelvic examinations revealed left lower tenderness with guarding. Laboratory investigations were within normal. Abdominal Doppler showed a dilated left ovarian vein with absent flow and Computed tomography (CT) scan confirmed the presence of a thrombus. Therefore, the diagnosis of OVT was made and she was started on anticoagulation.

**Discussion:**

OVT is a rare, life-threatening condition occurring mainly during the post-partum period (0.18% post vaginal delivery). Diagnosing ovarian vein thrombosis can be challenging because of the overlapping presentation with other differentials. A high index of suspicion should be kept in females presenting with abdominal pain. Standard guidelines for managing OVT are lacking. However, lower limb DVT guidelines have been suggested in the literature to be applicable.

**Conclusion:**

The initial modality of diagnosing OVT is Doppler ultrasound. However, CT and MRA confirm the diagnosis in case of uncertainty. The mainstay of treatment is the conservative approach while the surgical approach is reserved for persistent OVT despite appropriate conservative therapy.

## Highlights

• Ovarian vein thrombosis is rare, but a life-threatening condition.
• It is rarer in non-pregnant women and in patients without history of recent pelvic surgery as the case in our patients.
• The most commonly involved vein is the right ovarian vein, although in our case the left ovarian vein was affected.
• Diagnosis of ovarian vein thrombosis can be by CT scan or MRI but the initial modality should be doppler ultrasound.

## 1 Introduction

This work has been reported in line with the SCARE criteria [1].

Abdominal pain is one of the common clinical challenges presenting to the emergency department. The challenge lies in the wide differential diagnoses particularly in female patients due to the addition of potential gynecological conditions such as ovarian torsion, ovarian cyst and ovarian vein thrombosis. Ovarian vein thrombosis (OVT), if not diagnosed or treated early, can be complicated by sepsis and pulmonary embolism in 25% of the cases, which increase the mortality rate up to 4% [2].

## 2 Case presentation

**Fig. 1:**
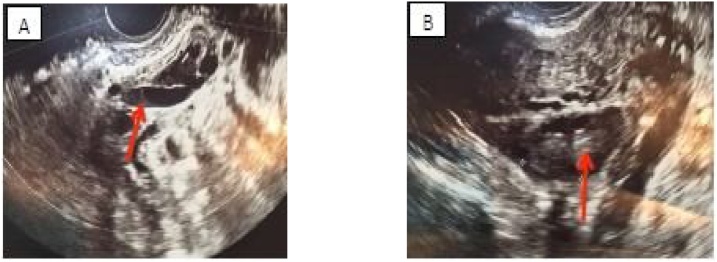
(A & B): Trans-Abdominal Ultrasound of the left ovary showing a dilated left ovarian vein.

**Fig. 2:**
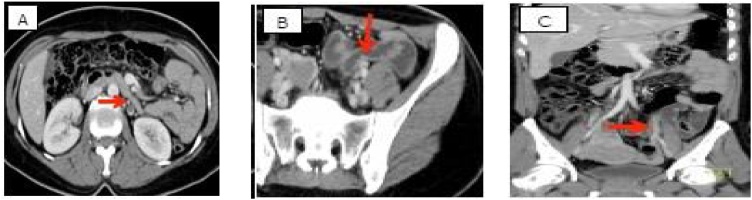
(A,B&C): CT scan with contrast of the Abdomen showing left ovarian vein thrombosis.

**Fig. 3:**
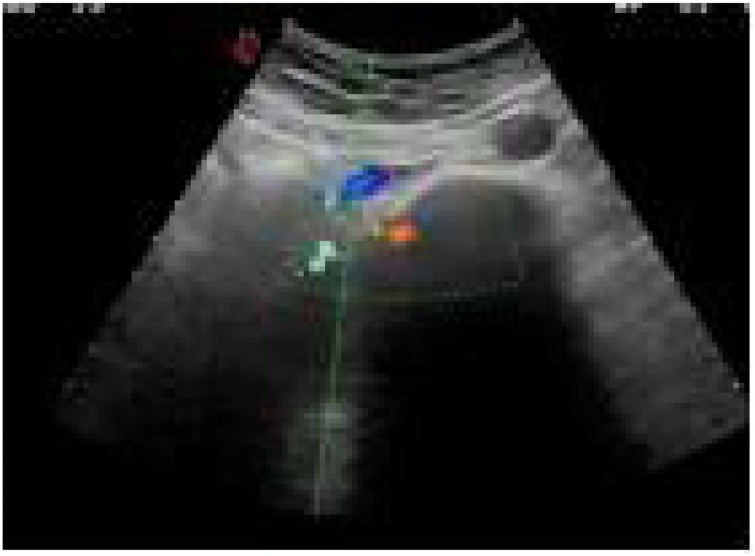
(A): Doppler ultrasound showing complete resolution of the thrombus.

Herein, we report a case of a 42-year-old female, medically free, with 5 previous normal vaginal deliveries, last delivery was 1 year prior to presentation. She was in her usual state of health until she presented to our emergency department complaining of sudden left iliac fossa and periumbilical pain associated with nausea and constipation for 1-day. The pain was not radiating with no aggravating or relieving factors. There was no fever, dysuria, hematuria or vaginal discharge. Her menstrual period was regular. She had no history of hypercoagulability or previous thromboembolic events as well as no history of previous surgery. Upon examination, she was afebrile & hemodynamically stable. Abdominal examination revealed left lower tenderness with guarding, with no palpable masses nor organomegaly. Her pelvic examination was unremarkable. Laboratory investigations showed no leukocytosis, negative C-reactive protein & Beta human chorionic gonadotropin with normal coagulation profile & a negative urine analysis. Transabdominal ultrasound of the left ovary showed dilated left ovarian vein (Fig. 1 A & B). Doppler abdominal ultrasound showed a dilated left ovarian vein with absent flow. Computed Tomography (CT) scan with contrast was done and revealed a thrombus in the left ovarian vein (Fig. 2A–C). Therefore, the diagnosis of OVT was made and the patient was started on low molecular weight heparin followed by warfarin for 6 months and discharged home after 1 week. She was followed up regularly for 1 year by Doppler ultrasound which showed complete resolution of the thrombus (Fig. 3).

## 3 Discussion

Ovarian vein thrombosis (OVT) is a rare yet, a life-threatening condition. It occurs mainly during the post-partum period with an incidence of 0.18% post normal vaginal delivery & in 2% after caesarian section [2]. Furthermore, the incidence increases even more with twin's delivery [4]. The association of ovarian vein thrombosis with post-partum period is related to the hypercoagulability status due to the surge of estrogen during pregnancy in addition to the compression of the gravid uterus on the ovarian vein. OVT can also develop post pelvic surgeries [5]. OVT usually presents with fever, pelvic pain, and right-sided abdominal mass in pregnant patients [3]. However, in our case, the patient was not pregnant, afebrile, no palpable mass was detected & did not undergo any previous surgeries. Diagnosing OVT can be challenging because of the overlapping presentation with other differential diagnoses like acute appendicitis, ovarian torsion or inflammatory bowel disease. Nevertheless, a high index of suspicion should be kept in patients presenting with abdominal pain. Doppler ultrasound should be the first radiological modality used to diagnose OVT due to its availability. However, it is inadequate to examine the whole length of the ovarian vein which necessitates the need for further imaging modalities [6]. The other available imaging modalities with better yield are CT with contrast & MRA, with the latter being the most sensitive & specific. However, contrast CT is both time & cost-effective choice [6]. The most commonly involved vein is the right ovarian vein in up to 90% of the cases. This could be attributed to its longer course, uterine dextroversion & the relative incompetency of the right ovarian vein valves [7]. The mainstay of managing OVT is usually conservative therapy in the form of antibiotics & anticoagulants. Yet,

gold standard guidelines are lacking. However, Wysokinska et al. suggested that lower limb DVT guidelines are applicable [8,9]. The duration of using anticoagulants depends on whether the cause of the thrombus is transient or persistent. The recommended use of anticoagulants in the literature ranges from 3 to 6 months and for those with hypercoagulable disease to be a life-long therapy. The American Society of Hematology recommend the use of anticoagulants for 3 months in addition to antibiotics if infection is suspected. The American College of Chest Physicians suggest the use of Rivaroxaban for VTE and PE in the absence of malignancy [[9], [10], [11]] However, surgical intervention like inserting an inferior vena cava filter or surgical ligation of the ovarian vein might be necessary in persistent cases despite optimal conservative approach [[12]. The use of direct oral anticoagulants (DOAC) for venous thromboembolism (VTE) is common in childbearing period, however the toxicity of using direct oral anticoagulant in human is unknown. The DOAC SPCs recommendation is antagonistic toward using oral anticoagulant therapy in pregnancy and while breastfeeding, however patients may accidentally become pregnant while taking DOAC therapy. Recommendations suggests that all women in their childbearing period should receive counseling prior to DOAC treatment with emphasis on using contraceptive while using oral anticoagulants to limit the chance of unfavorable pregnancy. Regular follow up and adherence to medications are important for women to assess their response, however the use of DOAC should be restricted in breastfeeding women due to absence of evidence of safety, while individual risk factor for nonlactating postpartum women should be considered [13].

## 4 Conclusion

Although OVT is a rare condition occurring mainly in post-partum women, it should be kept in mind as a differential diagnosis for iliac fossa pain. Abdominal Doppler ultrasound should be done first to diagnose OVT, however, contrasted CT and MRA have a higher sensitivity and specificity. The mainstay of treatment is the conservative approach by using antibiotics (if indicated) and anticoagulants, while the surgical approach is reserved for patients with persistent OVT despite appropriate conservative therapy.

## Conflict of interest

None.

## Sources of funding

None.

## Ethical approval

Case reports are exempt from the need of IRP approval in our institute.

## Consent

Written informed consent was obtained from the patient for publication of this case report and accompanying images. A copy of the written consent is available for review by the Editor-in-Chief of this journal on request.

## Author contribution

**Literature review:** Riyadh Al Abbas, Abdullah AlQattan, Muntadhar Alalqam, Fatimah Almabyouq, Abdaljaleel Abualsaud.

**Data collection:** Riyadh Al Abbas, Fatima Almabyouq.

**Writing of manuscript & reviewing of the final version of the manuscript**: all authors.

## Registration of research studies

Not applicable.

## Guarantor

Muntadhar M. Alalqam.

## Provenance and peer review

Not commissioned, externally peer-reviewed.

## References

[1] Agha R.A., Borrelli M.R., Farwana R., Koshy K., Fowler A., Orgill D.P., For the SCARE Group (2018). The SCARE 2018 statement: updating Consensus Surgical CAse REport (SCARE) Guidelines. Int. J. Surg..

[2] Harris K., Mehta S., Iskhakov E., Chalhoub M., Maniatis T., Forte F., Alkaied H. (2012). Ovarian vein thrombosis in the nonpregnant woman: an overlooked diagnosis. Ther. Adv. Hematol..

[3] Virmani V., Kaza R., Sadaf A., Fasih N., Fraser-Hill M. (2012). Ultrasound, computed tomography, and magnetic resonance imaging of ovarian vein thrombosis in obstetrical and nonobstetrical patients. Can. Assoc. Radiol. J..

[4] Angelini M., Barillari G., Londero A.P., Bertozzi S., Bernardi S., Petri R. (2012). Puerperal ovarian vein thrombosis: two case reports. J. Thromb. Thrombolysis.

[5] Labropoulos N., Malgor R.D., Comito M., Gasparis A.P., Pappas P.J., Tassiopoulos A.K. (2015). The natural history and treatment outcomes of symptomatic ovarian vein thrombosis. J. Vasc. Surg. Venous Lymphat. Disord..

[6] Suleyman T., Gultekin H., Abdulkadir G., Tevfik P., Abdulkerim U.M., Ali A., Ismail K. (2008). Acute right lower quadrant abdominal pain as the presenting symptom of ovarian vein thrombosis in pregnancy. J. Obstet. Gynaecol. Res..

[7] Doherty K., New M. (2015). Idiopathic ovarian vein thrombosis in a nonperipartum patient. Obstet. Gynecol..

[8] Johnson A., Wietfeldt E.D., Dhevan V., Hassan I. (2009). Right lower quadrant pain and postpartum ovarian vein thrombosis. Uncommon but not forgotten. Arch. Gynecol. Obstet..

[9] Wysokinska E., Hodge D., Mcbane R. (2006). Ovarian vein thrombosis: incidence of recurrent venous thromboembolism and survival. Thromb. Haemost..

[10] (2017). Hematol. Am. Soc. Hematol. Educ. Prog..

[11] Naoum J., Mohsen A., Daher J., Eid T. (2018). Novel management of ovarian vein thrombosis: a case report. J. Saudi Pharm. Soc..

[12] Takach T.J., Cervera R.D. (2005). Ovarian vein and caval thrombosis. Tex. Heart Inst. J..

[13] Cohen H., Arachchillage D.R., Middeldorp S., Beyer-Westendorf J., Abdul-Kadir R. (2016). Management of direct oral anticoagulants in women of childbearing potential: guidance from the SSC of the ISTH. J. Thromb. Haemost..

